# Deformability of Tumor Cells versus Blood Cells

**DOI:** 10.1038/srep18542

**Published:** 2015-12-18

**Authors:** Josephine Shaw Bagnall, Sangwon Byun, Shahinoor Begum, David T. Miyamoto, Vivian C. Hecht, Shyamala Maheswaran, Shannon L. Stott, Mehmet Toner, Richard O. Hynes, Scott R. Manalis

**Affiliations:** 1 Department of Biological Engineering, Massachusetts Institute of Technology, Cambridge, MA; 2 Koch Institute for Integrative Cancer Research, Massachusetts Institute of Technology, Cambridge, MA; 3 Howard Hughes Medical Institute, Massachusetts Institute of Technology, Cambridge, MA; 4 Massachusetts General Hospital Cancer Center, Harvard Medical School, Boston, MA; 5 Department of Radiation Oncology, Massachusetts General Hospital, Harvard Medical School, Boston, MA; 6 Department of Surgery, Massachusetts General Hospital, Harvard Medical School, Boston, MA; 7 Department of Medicine, Massachusetts General Hospital, Harvard Medical School, Boston, MA; 8 Massachusetts General Hospital Center for Engineering and Medicine, Harvard Medical School, Boston, MA; 9 Department of Biology, Massachusetts Institute of Technology, Cambridge, MA; 10 Department of Mechanical Engineering, Massachusetts Institute of Technology, Cambridge, MA

## Abstract

The potential for circulating tumor cells (CTCs) to elucidate the process of cancer metastasis and inform clinical decision-making has made their isolation of great importance. However, CTCs are rare in the blood, and universal properties with which to identify them remain elusive. As technological advancements have made single-cell deformability measurements increasingly routine, the assessment of physical distinctions between tumor cells and blood cells may provide insight into the feasibility of deformability-based methods for identifying CTCs in patient blood. To this end, we present an initial study assessing deformability differences between tumor cells and blood cells, indicated by the length of time required for them to pass through a microfluidic constriction. Here, we demonstrate that deformability changes in tumor cells that have undergone phenotypic shifts are small compared to differences between tumor cell lines and blood cells. Additionally, in a syngeneic mouse tumor model, cells that are able to exit a tumor and enter circulation are not required to be more deformable than the cells that were first injected into the mouse. However, a limited study of metastatic prostate cancer patients provides evidence that some CTCs may be more mechanically similar to blood cells than to typical tumor cell lines.

Carcinoma cells that have escaped into circulation, known as circulating tumor cells (CTCs), have drawn increasing interest in recent years due to their potential in cancer prognosis as well as the information they hold regarding a patient’s tumors^1^,^2^. However, CTCs are rare in the blood, estimated at one CTC per billion blood cells, and universal properties with which to identify them remain elusive^3^,^4^.

The most commonly used methods for CTC isolation are based upon antibody detection of cell surface antigens. Since epithelial cells express epithelial cell adhesion molecule (EpCAM), whereas blood cells do not, EpCAM is used to enrich CTCs from blood samples. Platforms utilizing this strategy include the CellSearch system (Veridex), which employs ferrofluid nanoparticles coated with anti-EpCAM antibodies to capture the cells, as well as microfluidic devices that are coated with anti-EpCAM antibody, where captured CTCs can be analyzed with further imaging^3^,^4^,^5^,^6^,^7^. Although the number of cells captured based on EpCAM expression have been shown to possess prognostic value for some cancers, it is not known what role these EpCAM expressing cells have in metastasis and whether another non-EpCAM expressing population of CTCs may provide additional information^8^,^9^,^10^,^11^,^12^. In order to avoid biases in positively selecting for surface markers, negative depletion is a method by which white blood cells are removed by anti-CD45 antibodies, thereby enriching the blood sample for CTCs^13^. One of several platforms is the CTC-iChip, which removes red blood cells by size-dependent deterministic lateral displacement and removes white blood cells by labeling them with magnetic beads, targeting CD45 and CD15^14^,^15^. However, negative depletion methods do not yet achieve 100% purity so additional approaches to distinguishing CTCs from blood cells are still required^3^,^16^.

In contrast to molecular based strategies for identifying CTCs, relatively fewer approaches are available for isolating CTCs by their physical properties. Two examples include a filtration system known as Isolation by Size of Epithelial Tumor cells (ISET, Rarecells)^17^, and dean flow fractionation, which involves a spiral channel employing centrifugal forces^18^. However, distinguishing between cell sizes does not provide sufficient specificity toward the cells being retained since small CTCs (similar in size to most leukocytes) may be lost, while large leukocytes may be enriched for^4^,^6^.

One particular physical property of single cells that has been widely explored in the context of cell malignancy is deformability. Previous studies have employed various methods to probe the mechanical properties of cancer cells from cell lines or body fluids, demonstrating that highly metastatic cells are often more deformable than weakly metastatic cells^19^,^20^,^21^,^22^,^23^,^24^,^25^,^26^,^27^,^28^,^29^,^30^,^31^,^32^. However, to the best of our knowledge, no one has yet directly compared the deformability of CTCs to that of blood cells. In recent years, technology for measuring single-cell deformability has entered a stage where researchers can almost as easily measure the deformability as they can the size of single cells (Supplementary Table S1)^24^,^33^,^34^. Nonetheless, to achieve the level of being used to routinely analyze rare CTCs in patient blood, existing platforms would need further advancement. To assess whether this development is worthwhile, one must first determine if there are differences in deformability between blood cells and CTCs. If CTCs and blood cells do prove to possess distinct measures of deformability, it may suggest a valuable strategy for isolating CTCs.

Here, we use a suspended microchannel resonator (SMR) with a constriction to take initial steps towards characterizing differences in deformability between tumor cells and blood cells, based on the length of time required for each cell to pass through the constriction (Fig. 1). We first show that cancer cell lines are mechanically distinct from blood cells, and that the differences between cells having undergone phenotypic shifts are small when compared to the difference between such cell lines and blood cells. Next, we use a mouse model to assess the deformability of tumor cells once they enter into circulation, particularly noting their distinctions from the surrounding blood cells in their new environment. Finally, we measure the deformability of CTCs from a limited number of cancer patients and find evidence that the deformability of some CTCs can be similar to that of blood cells. Although the limited throughput of our approach combined with the rarity of CTCs prevents us from obtaining a definitive analysis, our findings suggest the need for future studies and a deeper consideration of the deformability of CTCs.

## Results

### Deformability Changes undergone by Tumor Cells in Different States

**Figure 2:**
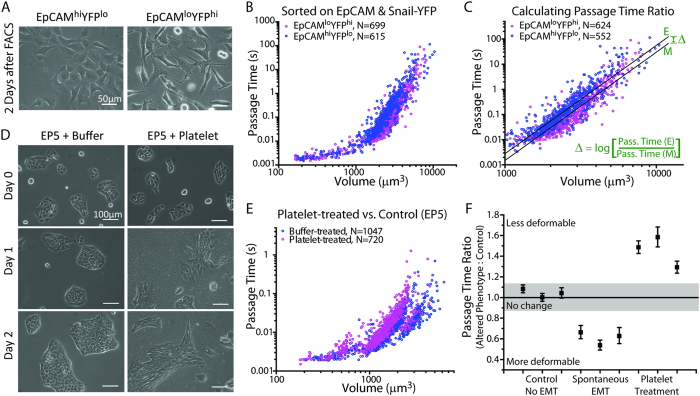
Deformability changes in tumor cells having undergone a phenotypic shift. (**A**) FACS sorted epithelial (EpCAM^hi^YFP^lo^) and mesenchymal (EpCAM^lo^YFP^hi^) populations of the MMTV-PyMT tumor cell line were cultured for two days to confirm differences in morphology prior to measurement in the SMR. Phase contrast micrographs indicate that the mesenchymal population has decreased intercellular adhesion and is more elongated than the epithelial population. (**B**) Passage time versus volume, comparing the epithelial and mesenchymal subpopulations of the MMTV-PyMT cell line. (**C**) A zoomed-in region of data from (**B**) with volumes greater than 1000 μm^3^, demonstrating a passage time ratio calculation, taking the difference in y-intercepts (Δ) as the exponent of 10 (Supplementary Methods). Here, the intercept offset is significantly different from zero (p < 2 × 10^−16^) based on the linear regression. (**D**) Phase contrast micrographs of control EP5 cells (treated with buffer) and EP5 cells treated with platelets. Cells were treated on day 0 after images were taken. Images taken on subsequent days reveal morphological changes in the platelet-treated cells undergoing an EMT-like transition. (**E**) SMR measurements of the buffer-treated EP5 cells and the platelet-treated EP5 cells after two days of co-incubation with platelets. (**F**) Passage time ratios were calculated for three replicates of each control experiment (different plates of buffer-treated EP5 cells compared to one another: Control, No EMT), three replicates of the MMTV-PyMT epithelial versus mesenchymal comparison (Spontaneous EMT), and three replicates of the buffer-treated versus platelet-treated EP5 cell comparison (Platelet Treatment). Each passage time ratio represents the passage time of the cells with an altered phenotype (EMT or co-incubation with platelets) to that of the control population. In both comparisons of mesenchymal or platelet-treated cells to control cells, the y-intercepts of the linear fits were found to be significantly different (p < 3.4 × 10^−16^). Error bars for each point correspond to the 95% confidence interval of the passage time ratio based on the intercept offsets of the linear fits. The shaded region is based on the extent of change seen in control experiments, and is a symmetric region drawn about a passage time ratio of 1.0, indicating no change.

As tumor cells may exist in various states during the metastatic process, we began by measuring changes in deformability between different tumor cell phenotypes. One example of tumor cells having altered phenotypes is in an epithelial-mesenchymal transition (EMT), where cells decrease cell-cell adhesion and increase their motility and invasiveness^35^,^36^,^37^. The EMT program has been identified in a pancreatic ductal adenocarcinoma mouse model to precede frank tumorigenesis, and has been implicated in human carcinoma biopsies as well as in CTCs of breast cancer patients^38^,^39^,^40^. Here, we measure the deformability difference (Methods) in a spontaneous EMT undergone by a murine tumor cell line derived from a mammary carcinoma of a *Snail*^YFP^/+; *MMTV-PyMT* animal^41^. In this cell line, a YFP reporter is directly controlled by the endogenous promoter of the EMT-inducing transcription factor *Snail*, which allows fractionation of the cells according to *Snail* expression levels. This cell line was sorted using fluorescence-activated cell sorting (FACS) into an epithelial population (EpCAM^hi^YFP^lo^) and a spontaneously arising mesenchymal (EpCAM^lo^YFP^hi^) population. After sorting, the cells were propagated in monolayer culture for a few days, and differences in epithelial and mesenchymal morphologies were confirmed by phase-contrast microscopy (Fig. 2A). The two cell populations were then trypsinized and measured separately in the SMR, revealing that for cells of the same size, the cells of the mesenchymal population generally pass through the constriction faster than do those of the epithelial population (Fig. 2B). As previously described^28^, lines were fit to each data set such that the difference in y-intercepts determines the ratio of the passage time of the mesenchymal population to that of the epithelial population, given the same cell size (Fig. 2C, Supplementary Methods). In addition, for such close comparisons, buoyant mass values were converted to volume by measuring the density of the cells in the SMR (Supplementary Methods). Replicates and passage time ratio values are plotted in Fig. 2F, showing that on average, the EpCAM^lo^YFP^hi^ cells passed through the constriction 1.6-fold faster than the EpCAM^hi^YFP^lo^ cells, for those having a volume greater than 1000 μm^3^. It is also already known that the EpCAM^lo^YFP^hi^ population of cells is more than two-orders of magnitude more tumorigenic than the EpCAM^hi^YFP^lo^ population when injected into mice^41^. This result is consistent with studies by other methods demonstrating that often, more mesenchymal or malignant cell types are more deformable^19^,^20^.

Next, platelets have been shown to interact with tumor cells, correlate with increased metastasis, and induce an epithelial-mesenchymal-like (EMT-like) transition in tumor cells^42^,^43^,^44^,^45^,^46^. Hence, as another case study of deformability changes undergone by tumor cells, we measured the deformability changes in tumor cells induced by co-incubation with platelets, using a previously studied model system where the molecular and cell-biological changes are already known^45^. The published study demonstrated that after one day of co-culture with platelets, EP5 murine breast carcinoma cells already exhibit increased expression of EMT-related, and prometastatic genes. Following an additional day of co-culture, the carcinoma cells, having the majority of the platelets washed away during sample preparation, generate an increased number of lung metastases after tail-vein injection^45^. Using the same cell line and technique as previously published^45^, we cultured EP5 cells with platelets for two days, and confirmed that the cells possessed decreased intercellular adhesion and elongated morphology (Fig. 2D) as specified in the previous study^45^. While one culture plate of cells was treated with platelets, two control plates were treated with an equal amount of buffer. Cells from all plates were then washed, rinsing away platelets, and measured separately in the SMR to compare any changes in deformability between the platelet-treated and buffer-treated cells, using the deformability difference between the two buffer-treated plates as a control (Fig. 2E, Supplementary Fig. S1). For the EP5 platelet-treated and buffer-treated comparisons, the population of cells having a larger volume (Fig. 2E and Supplementary Fig. S1) is presumed to be doublets that did not dissociate after trypsinization, since those cells have twice the volume of the majority of cells, and hence were not included in the linear fits when determining the passage time ratios (Supplementary Methods, Supplementary Fig. S2). Interestingly, the platelet-treated cells were not more deformable than the control cells; rather, they took a longer time to pass through the constriction in every replicate (Fig. 2F). On average, the platelet-treated EP5 cells required a 1.5-fold longer time than the control cells to pass through the constriction, for cells between 800 μm^3^ and 3500 μm^3^. The deformability changes in tumor cells caused by signaling from platelet interactions have not been measured previously, and the decreased deformability brought about by this interaction differs from most studies referenced earlier that indicate that more malignant cells are often more deformable. As postulated by others, the decreased deformability may enhance hematogenous dissemination by augmenting survival under shear stress conditions and increasing the ability of the cells to lodge or be retained longer in microcirculation^27^,^46^,^47^,^48^. Overall, the changes in passage time related to phenotypic or morphological changes in EMT or by co-culture with platelets are similar in magnitude.

### Passage Time Differences between Tumor Cell Lines and Blood Cells

**Figure 3:**
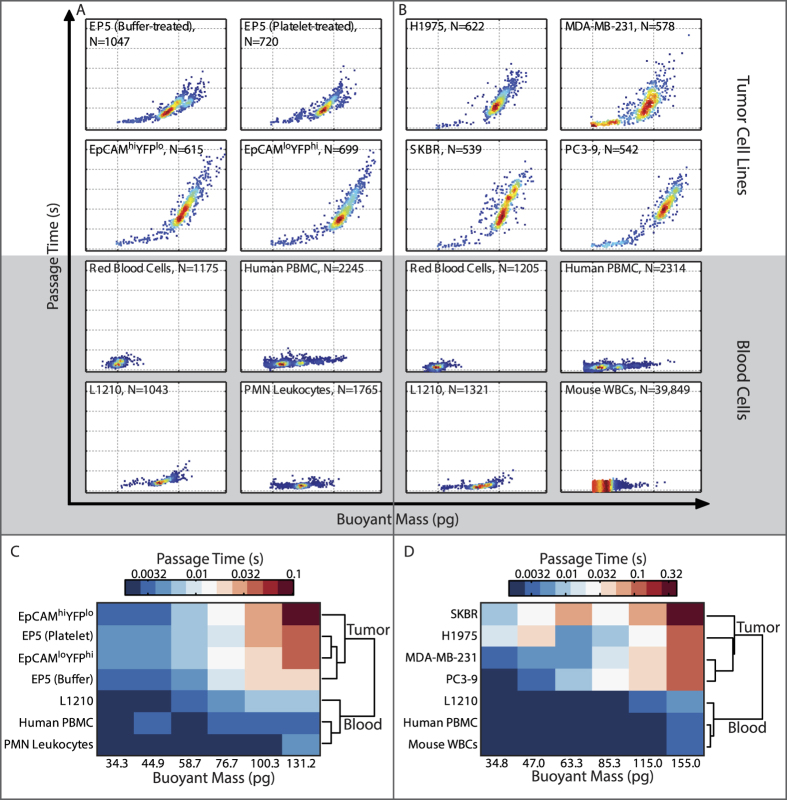
Passage time and buoyant mass of blood cells versus epithelial and mesenchymal cell lines. Passage time versus buoyant mass plots demonstrate the distinct difference between tumor cell lines and blood cells, regardless of the epithelial or mesenchymal phenotype of the tumor cells. (**A**) The same murine cell lines (EP5, EpCAM^hi^YFP^lo^, EpCAM^lo^YFP^hi^) from Fig. 2 are shown here in comparison with blood cells: human red blood cells, human peripheral blood mononuclear cells (PBMC), human polymorphonuclear (PMN) leukocytes, and a mouse lymphoblast cell line (L1210), measured under the same flow conditions (0.9 psi applied pressure). (**B**) Four human cancer cell lines corresponding to lung (H1975), breast (MDA-MB-231, SKBR), and prostate cancers (PC3-9) compared to blood cells: human red blood cells, human PBMC, mouse white blood cells (WBCs) and L1210 cells, under faster flow conditions (1.5 psi applied pressure) portray that the same distinction in passage time profiles. For (**A**,**B**), the dotted grid lines on the X-axis are at 10 pg and 100 pg, while dotted grid lines on the Y-axis are at 0.001 s, 0.01 s, 0.1 s, 1 s, 10 s, and 100 s. Also, the color of the scatter plots corresponds to the density of data points, with red being the highest and blue being the lowest density. (**C**) The passage time data in (**A**) for buoyant masses between 30 pg and 150 pg are placed into 6 bins based on buoyant mass (log scale). The median of the log_10_ values of the passage times within each bin is shown in the heat map, where color assignment is on a log scale. The passage times are grouped using agglomerative clustering, with the dendrogram shown to the right of the heat map. The reported buoyant mass bin centers and passage time values are converted from log_10_ values. (**D**) Similar to (**C**), the passage time data in (**B**) for buoyant masses between 30 pg and 180 pg are plotted in a heat map of passage time values, showing a dendrogram of the clustering to the right.

After identifying possible deformability changes within tumor cell populations, we compare tumor cell populations to blood cells. During the process of metastasis, it is thought that some tumor cells enter into and survive in circulation, leading them to a new site where they may form metastatic lesions^49^. Thus, to begin probing the biophysical differences between cells that naturally live and survive in circulation versus ones that typically live within a tissue parenchyma, we first compared the passage time and buoyant mass properties of tumor cell lines versus blood cells. Figure 3 shows distinct size and deformability characteristics between these two types of cells (top two rows versus bottom two rows of Fig. 3A,B), where the small differences between mesenchymal and epithelial tumor cell phenotypes pale in comparison to the dramatic differences between the tumor cell types and blood cells. Indeed, blood cells have passage times on the order of a few milliseconds, whereas tumor cells can have passage times as long as a few seconds. Included in the various tumor cell lines measured are a lung cancer cell line (H1975), two breast cancer lines (MDA-MB231 and SKBR-3), and a prostate cancer line (PC3-9). Meanwhile, the types of blood cells measured include human erythrocytes, peripheral blood mononuclear cells (PBMC), polymorphonuclear (PMN) leukocytes, L1210 (mouse lymphoblast cell line), and primary mouse (BALB/c) leukocytes (Supplementary Methods).

As is already known, the majority of blood cells are smaller than most of the tumor cells in culture. However, even for cells of comparable size, as indicated by their buoyant mass, blood cells have decidedly faster passage times than do tumor cells. This distinction holds true for passage time measurements at two different flow rates, as portrayed by the similarity between Fig. 3A,B. For each flow condition, agglomerative clustering was employed on the median of the log_10_ values of the passage times for binned buoyant masses, given a set range of buoyant masses where the different cell types overlapped (Methods). The unsupervised algorithm demonstrates that the data naturally fall into two separate groups: tumor cells and blood cells (Fig. 3C,D). Moreover, it is worth noting that both cell types were measured in a suspended state; thus even after trypsinization and resuspension in culture medium, the tumor cells maintain a marked physical distinction from blood cells. Given such a contrast, the question arises as to whether the circulatory system requires cells to possess a specific deformability characteristic in order for them to intravasate and remain in circulation.

### Passage Time Differences between Tumor Cells and Blood Cells in Circulation in a Mouse Model

**Figure 4:**
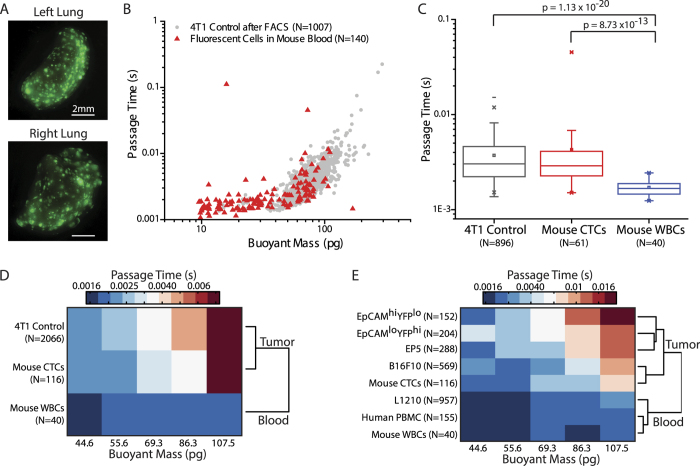
Mouse CTC deformability. (**A**) Mouse lungs were imaged seven days after tail vein injection of one million 4T1-ZSGreen cells to confirm presence of metastatic lesions. (**B**) Mouse blood, after lysing the erythrocytes, was run through FACS to sort out tumor cells having green fluorescence, and then measured in the SMR. The red triangles indicate each signal detected by the SMR while measuring CTCs. 4T1-ZSGreen control cells kept in culture after the initial injection were run through FACS for a comparison, and are shown as gray points in the background. (**C**) The distributions of passage times for the 4T1-ZSGreen control cells, CTCs, and BALB/c leukocytes (same data as in Fig. 3B) are plotted for cells with a buoyant mass ranging from 40 pg to 120 pg, eliminating debris, aggregates, or stray blood cells sorted during FACS while focusing on cells large enough to interact with the constriction. The control cell line and the CTCs have similar passage time distributions (p = 0.412). P-values were obtained from two-sided Wilcoxon rank-sum tests. (**D**) Data from three replicates of the experiment are pooled to compare passage times in the given mass range. The heat map colors correspond to the passage time value, with the color assignments on a log scale. The binned passage time values were grouped by agglomerative clustering, with the dendrogram shown to the right of the heat map. (**E**) CTC data from three replicates are pooled and compared to murine tumor cell lines other than the control 4T1 cell line, including EP5, EpCAM^hi^YFP^lo^, EpCAM^lo^YFP^hi^, and B16F10, evaluated under the same flow conditions (1.5 psi applied pressure). Also included for comparison are the blood cells from Fig. 3B. The passage times are plotted in the heat map and the dendrogram from clustering analysis is shown on the right. For (**D**,**E**), the reported buoyant mass bin centers and passage time values are converted from log_10_ values.

To assess tumor cells circulating *in vivo*, we injected one million 4T1-ZSGreen (a murine mammary carcinoma line stably transfected to express ZSGreen) cells via tail vein into syngeneic BALB/c mouse hosts. 4T1 cells are known to express epithelial markers such as E-cadherin, while also expressing Twist, an EMT-related transcription factor^50^,^51^. In addition, they are known to be migratory, invasive, and highly metastatic^50^,^51^. After one week, metastatic lesions had formed in the lungs (Fig. 4A) and blood was retrieved from the mouse via cardiac puncture. Tumor cells were then sorted from the blood using FACS, based on their ZSGreen expression and subsequently measured in the SMR. The measured cells from the blood were compared to the 4T1 cells that had been injected seven days prior, an aliquot of which had been kept in culture and run through FACS as a control (Fig. 4B, Supplementary Fig. S3).

Tumor cells found to persist in the blood seven days after initial intravenous injection are expected to be cells that had detached from initially formed metastatic tumors and re-entered circulation, since the originally injected cells should have all been removed from circulation within seven days, if not within a few minutes^52^,^53^. In each of three replicates of the experiment, over 97% of the initially injected cells maintained in culture, spanned the same range of passage times as the tumor cells that were able to enter into the blood stream from the metastatic lesions. In fact, for the one replicate having more than 40 CTCs in the size region of interest (40 pg to 120 pg), the distribution of passage times for CTCs is not significantly different from that of the original 4T1 control cell line (two-sided Wilcoxon rank-sum test, p = 0.412, Fig. 4C). Therefore, although the cells may transiently change their deformability during intravasation, our measurement reveals that there is not a strict deformability requirement that the cells must meet in order to enter circulation. Additionally, both the CTCs and the control cell line have higher median passage times (1.7- and 1.8-fold, respectively) than healthy BALB/c leukocytes, with the passage time distributions of the tumor cells being significantly different from that of the blood cells. Due to the small number of CTCs in other replicates, rather than assessing the distribution of passage times, agglomerative clustering of median passage times was employed as in Fig. 3, combining the data from all replicates (Fig. 4D,E). As a result, the mouse CTCs cluster with the control 4T1 cells and not with the leukocytes (Fig. 4D). In addition, the CTCs also cluster with other murine cell lines measured under the same flow conditions, such as EP5, EpCAM^hi^YFP^lo^, EpCAM^lo^YFP^hi^, and B16F10 melanoma cells, instead of grouping with blood cells, including L1210, human PBMCs, and healthy BALB/c leukocytes (Fig. 4E, Supplementary Fig. S4). The data from individual replicates also separately cluster in the same fashion, grouping themselves with other tumor cells rather than blood cells (Supplementary Fig. S5). Hence, cells that intravasate into the blood stream do not necessarily need to be as deformable as blood cells. It is noteworthy that based on the passage time characteristic alone, not taking into account any molecular differences, a cell having almost any measureable passage time from the original 4T1 population could intravasate into the blood stream, even though its passage time can be 10-fold higher than that of typical leukocytes.

### An Initial Study of the Passage Times of Cancer Patient CTCs compared to those of Blood Cells

**Figure 5:**
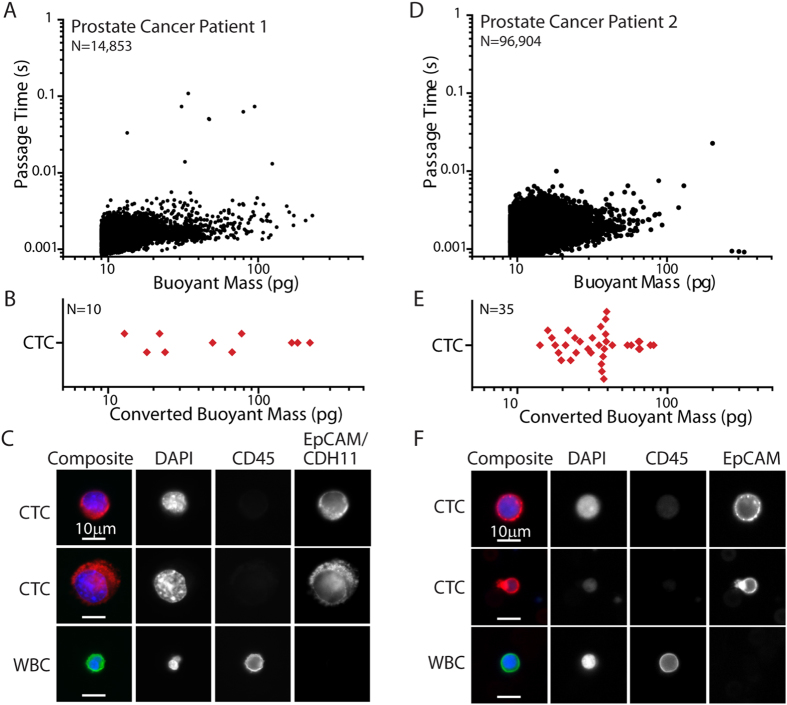
Human patient CTC deformability. (**A**) SMR measurement of prostate cancer Patient 1 blood sample, after having been processed in the CTC-iChip^14^,^15^. (**B**) Based on fluorescence images, the diameter of each CTC was converted to an approximate buoyant mass value to visualize where they fall among the data measured by the SMR as shown in (**A**). Each point represents one cell. (**C**) A sampling of fluorescence images of CTCs and a white blood cell (WBC) taken from the same prostate cancer patient sample after it was measured in the SMR. (**D**) SMR measurement of prostate cancer Patient 2 blood sample, after having been processed in the CTC-iChip. (**E**) Based on fluorescence images, the diameter of each CTC was converted to an approximate buoyant mass value. Each point represents one cell. (**F**) A sampling of fluorescence images of CTCs and a WBC from the same patient sample. False color overlays were applied for composite fluorescence images.

We subsequently turned to human patient samples to assess whether tumor cells in cancer patients also maintain a passage time in the circulation distinct from that of blood cells. Blood samples from metastatic prostate cancer patients were processed with the CTC-iChip at the Massachusetts General Hospital, depleting the samples of the majority of red blood cells and white blood cells as previously described^14^,^15^. Although the concentration of CTCs produced by the CTC-iChip helped enable the measurement of some CTCs by the SMR in spite of its low throughput (45 μL/hr), most samples did not contain EpCAM positive CTCs in the sample volume measured by the SMR. Thus, only limited observations are reported here. The two samples processed having multiple CTCs are shown in Fig. 5. As expected, due to residual blood cells in the samples, most of the cells passed through the SMR’s constriction quickly, doing so without any apparent hindrance. However, to verify whether CTCs were also measured, all cells were collected from the output of the SMR and stained with DAPI for the nucleus, EpCAM to indicate a tumor cell^54^, and counterstained for CD45 as a marker for leukocytes (Methods). To then determine whether there were CTCs that passed through the SMR’s constriction with a timescale comparable to that of blood cells (<0.01 s), we compared the number of CTCs (DAPI positive, EpCAM positive, and CD45 negative) to the number of long passage time events (>0.01 s). For both patients, there were more CTCs than long passage time events (one more for Patient 1, 34 more for Patient 2), indicating that CTCs can behave like blood cells in terms of passage properties.

However, since it is known that CTCs can be small^6^,^14^ and that most cells below 50 pg pass through the SMR’s constriction in less than 0.01 s regardless if they are deformable blood cells or potentially stiffer adherent cells placed into suspension (1.5 psi applied pressure, Fig. 3B), estimates were made of the buoyant mass of each CTC based on the imaged diameter (Supplementary Methods, Supplementary Figs S6 and S7). The buoyant mass of each CTC was not known directly since, although they were measured by the SMR, the identity of each individual cell was not inherently known during the measurement. From the buoyant mass conversion, many CTCs were in fact small compared to the cells prepared from cultured cancer cell lines and measured previously (Fig. 3). For this reason, these CTCs were below the sensitivity range of the SMR to definitively compare their deformability to that of blood cells. However, there were some large CTCs that would be expected to have passage times greater than 0.01 s if they were mechanically similar to those deriving from a tumor cell line. Based on a combination of all the human tumor cell line data in Fig. 3B (Methods), a tumor cell between 50 pg and 100 pg has a probability of 0.58 of having a passage time greater than 0.01 s, and a tumor cell greater than 100 pg has a probability of 0.99 of having a passage time greater than 0.01 s. For Patient 1, there were three CTCs greater than 100 pg, while there was only one SMR measurement having a passage time greater than 0.01 s. If the CTCs are like the tumor cell lines, the probability of only one of the three CTCs having a passage time above 0.01 s is 3.0 × 10^−4^. Similarly, for Patient 2, there were 5 CTCs between 50 and 100 pg, and the probability of all of them having passage times faster than 0.01 s to match the SMR measurements is 0.013. Thus, it is unlikely that all of the larger CTCs in these patient samples are physically similar to the tumor cell lines for cells of the same size; rather some of them more closely resembled blood cells in their ability to pass through the constriction quickly.

## Discussion

In considering tumor cells in circulation, we have found that in spite of deformability changes due to an EMT or co-incubation with platelets, tumor cells in general (whether having an epithelial or mesenchymal phenotype) have much longer passage times than blood cells. Furthermore, based on the mouse model presented, there does not seem to be a specific deformability necessary for the 4T1 tumor cells to exit the initially formed metastases and enter circulation. More specifically, the tumor cells that enter circulation from the metastatic lesions are not particularly more deformable than the originally injected population of tumor cells. This observation is consistent with the fact that during invasion and intravasation, the surrounding extracellular matrix can be proteolytically degraded^55^ and endothelial cell junctions may be weakened^56^, possibly allowing tumor cells to pass through without requiring them to be especially deformable.

In human prostate cancer patients, however, the throughput limitations of the SMR device required patients to have high CTC levels. Based on two patient samples having CTCs, many of their CTCs were able to pass through the constriction in less than 0.01 s, as fast as blood cells. Part of this phenomenon is due to the small size of many CTCs. However, if CTCs were mechanically similar to the various tumor cell lines we studied, it would be unlikely for the larger CTCs to also have fast passage times as were seen in the SMR measurements. Thus, while the mouse model demonstrates that blood-cell-like deformability is not necessary for some tumor cells to intravasate and enter circulation, limited human patient samples show that some of the CTCs in circulation may in fact find it advantageous to be small in size or to be as flexible as blood cells.

The observation of deformable CTCs, of course, does not preclude the existence of other, stiffer CTCs in the circulation having longer passage times, similar to those of cells from tumor cell lines. The current approach only provides evidence for the presence of deformable, EpCAM-expressing CTCs, since the number of cells expressing EpCAM exceeded the number of cells having long passage times. In order to additionally confirm the presence of CTCs having long passage times, the measurement technique would require a single-cell sorting method to determine the correlation between the molecular expression and biophysical characteristic of each cell individually. Certainly, the limited data presented here would need to be much expanded and developed to identify the range of the deformability of CTCs relative to blood cells. However, due to the low throughput of the SMR combined with the rarity of CTCs, further enhancements to the platform, such as increased throughput via parallelization, would need to be implemented to make a more complete study feasible. In addition, exploring the effects of various constriction sizes as well as utilizing other platforms to assess cell deformability at a broader range of timescales, or shear rates, may aid in more definitively defining the mechanical distinguishability between CTCs and blood cells. Taken together, our findings suggest that the deformability of CTCs may not be as straightforward as commonly thought, as many researchers use tumor cell lines as a surrogate for CTCs, and that more in-depth studies using a variety of platforms may be necessary to fully reveal the range of mechanical properties of patient CTCs.

## Methods

### Single Cell Deformability and Size Measurements

A suspended microchannel resonator (SMR) having a 6 μm wide, 15 μm deep, and 50 μm long constriction was used to measure the buoyant mass, as a metric for size, as well as the passage time, as a metric for deformability, of each single cell as previously described^28^. In addition to deformability, the passage time measurement is also influenced by such factors as cell size and surface friction^28^. Therefore in all analyses of deformability, cells of the same size were compared. For passage time ratios and cluster analyses, size regions of interest were chosen to ensure overlap in buoyant mass or volume between the cell types being compared, eliminate extraneous debris or aggregates, and focus on cells large enough to interact with the constriction. Moreover, the relative contribution of surface friction to the passage time can be accounted for by the cell’s velocity as it transits through the constriction (transit velocity), after fully deforming into its entrance^28^. However, the velocity of the cell upon entering the constriction (entry velocity) is typically slower than the transit velocity, suggesting that the passage time is mostly dictated by the time the cell spends deforming at the entrance of the constriction, rather than transiting through afterwards (Supplementary Fig. S8). The transit velocity of cells undergoing an EMT or co-incubated with platelets was found to change by the same amount as the entry velocity, indicating that alterations in the frictional component affect but do not dominate the differences in passage times (Supplementary Fig. S8). Hence, in the data presented here, we consider changes in passage times to dependably represent changes in deformability, although frictional roles are also included in the holistic passage time measurement. Also, for cells involved in an EMT or co-incubation with platelets, where the change in passage time may be subtle, buoyant mass was converted to volume by measuring the density of each cell type (Supplementary Methods). The channel walls were coated with PEG (1 mg/mL; PLL(20)-g[3.5]-PEG(2); Surface Technology) for all experiments in this study. All SMR data was processed in MATLAB as previously described^28^, and passage time density scatter plots were created using a modified version of the dscatter function (MathWorks File Exchange, ref. 22).

### Cluster Analysis of Passage Time Data

For data compared by clustering analysis, log_10_ values of the buoyant mass and passage time data were used, both in binning buoyant mass values and determining median passage times. Thus, the passage time value reported for each buoyant mass bin is the median of the log_10_ values of the passage times converted back to an actual time by taking it as an exponent of 10. The reported buoyant mass bin centers were also converted from log_10_ values by exponentiation. The agglomerative hierarchical clustering analysis was performed using MATLAB.

### Platelet Preparation and Treatment of EP5 Cells

Platelets were prepared and EP5 cells were co-cultured as previously described^45^. Platelets were resuspended in PIPES buffer (1,4-piperazinediethanesulfonic acid, Sigma-Aldrich), and at least 500 million platelets were used to treat each 6-cm dish of EP5 cells. Buffer-treated cells were treated with the same volume of PIPES buffer, but without platelets. When the cells were ready to be measured in the SMR, they were washed with PBS, trypsinized, and resuspended in culture medium.

### Mouse CTC Generation and Preparation

All experiments involving mice were performed under the approval of the Committee on Animal Care at the Massachusetts Institute of Technology. All methods were carried out in accordance with the approved guidelines. Balb/c mice were injected via tail vein with 1 million 4T1-ZSGreen cells in 100 μL of Hank’s Balanced Salt Solution (HBSS, Life Technologies). After seven days, blood was collected by cardiac puncture (left ventricle) into 400 μL of citrate dextrose solution (ACD; 38 mM citric acid, 75 mM trisodium citrate, 100 mM dextrose). Fluorescence images were taken of the lung to verify tumor formation. Meanwhile, red blood cells were lysed using lysis buffer for 2 minutes on ice. Cells were then resuspended in phosphate buffered saline (PBS) with 0.2% FBS and prepared for FACS. Cells were collected into culture medium, filtered with a 70 μm mesh, and subsequently measured in the SMR.

### Human Patient CTC SMR Measurements and Staining

Patients with a diagnosis of prostate cancer provided informed consent to an Institutional Review Board approved protocol (DF/HCC 05-300) at the Massachusetts General Hospital (MGH) to allow donation of blood for this study. All methods were carried out in accordance with the approved guidelines. Negative depletion was performed on the patient samples using the CTC-iChip as previously described^14^,^15^. Samples were maintained at room temperature in PBS with 1% Kolliphor P188 during SMR measurement. For Patient 1, measured cells were placed in a 24-well glass bottom plate coated with Cell-tak (BD Biosciences 354240). Paraformaldehyde (PFA) was then added to each well to reach a final concentration of 4% in each well (PFA was made fresh from 16% PFA, Electron Microscopy Sciences 15710). The well-plate was then spun at 821 rcf for 10 min to ensure adherence of the cells. After over 30 min of fixation, the cells were permeabilized with 1% Igepal (Sigma-Aldrich), rinsed, and incubated with 3% bovine serum albumin (BSA, Fisher) and 5% mouse serum (Jackson ImmunoResearch 015-000-120) in PBS. Cells were then stained with anti-Cadherin-11 and anti-EpCAM antibodies both conjugated to Alexa Fluor 488 (R&D Systems FAB17901G, Cell Signaling Technologies 5198S), anti-CD45 conjugated to PE-CF594 (BD Biosciences 562279), and DAPI (Life Technologies D1306). The cells were then rinsed and stored in PBS for fluorescence imaging. For Patient 2, after passing through the CTC-iChip, cells were stained with anti-EpCAM (Cell Signaling Technologies 7139) and anti-CD45 (Invitrogen MHCD4520) antibodies. Stained, live cells were then measured in the SMR and collected in a 24 well plate. PFA was added to a final concentration of 4%, along with DAPI (1.43 μM final concentration) for nuclear staining. Based on the concentration of the samples during negative depletion, an equivalent of approximately 1 mL of blood from Patient 1 was measured, and an equivalent of 1.6 mL of blood from Patient 2 was measured in the SMR.

### Calculating Passage Time Probabilities for Tumor Cells

Probabilities of the passage times of tumor cells based on their buoyant masses were calculated by pooling all of four sets of human tumor cell line data from Fig. 3B. For cells in a given size range, the fraction of cells having passage times greater than 0.01s was calculated as the probability used in the analysis described in the main text.

### Fluorescence Imaging of CTCs

Fluorescence imaging was done on a Nikon Ti inverted microscope (Swanson Biotechnology Center Microscopy Core Facility). All imaging of CTCs was performed at 40× magnification. Images were taken on a Photometrics CoolSnap HQ camera. The diameter of each cell was found by taking the maximum Feret’s diameter of the fluorescence EpCAM signal after background subtraction using ImageJ.

## Additional Information

**How to cite this article**: Shaw Bagnall, J. *et al.* Deformability of Tumor Cells versus Blood Cells. *Sci. Rep.* **5**, 18542; doi: 10.1038/srep18542 (2015).

## Supplementary Material

Supplementary Information

## Figures and Tables

**Figure 1:**
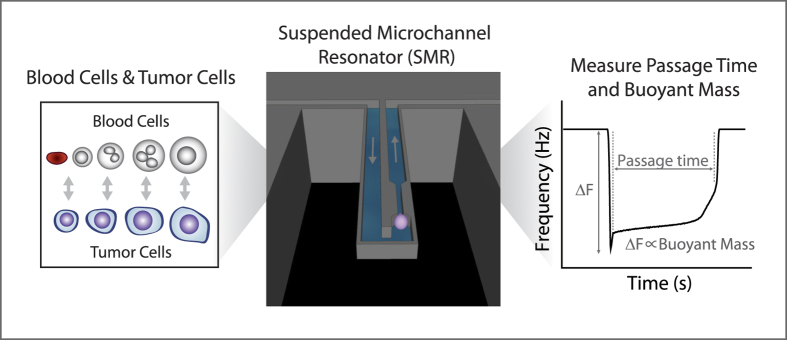
Measuring deformability differences between tumor cells and blood cells. The deformability of tumor cells is compared to that of blood cells of the same size. Single-cell deformability is measured by the SMR as a passage time, which is the amount of time it takes the cell to squeeze through a 6 μm wide microfluidic constriction. Simultaneously, as the cell traverses through the SMR cantilever, its buoyant mass is measured as a metric for cell size.
