# Supplementary material for: Deformability of Tumor Cells versus Blood Cells
Source: Sci Rep. 2015 Dec 18;5:18542. doi: 10.1038/srep18542 (PMC4683468; doi:10.1038/srep18542)
Supplement: Supplementary Information [file srep18542-s1.pdf]

Deformability of Tumor Cells versus Blood Cells

Josephine Shaw Bagnall*, Sangwon Byun*, Shahinoor Begum, David T. Miyamoto, Vivian C. Hecht, Shyamala Maheswaran, Shannon L. Stott, Mehmet Toner, Richard O. Hynes, Scott R. Manalis

*equal contribution

Supplementary Figures

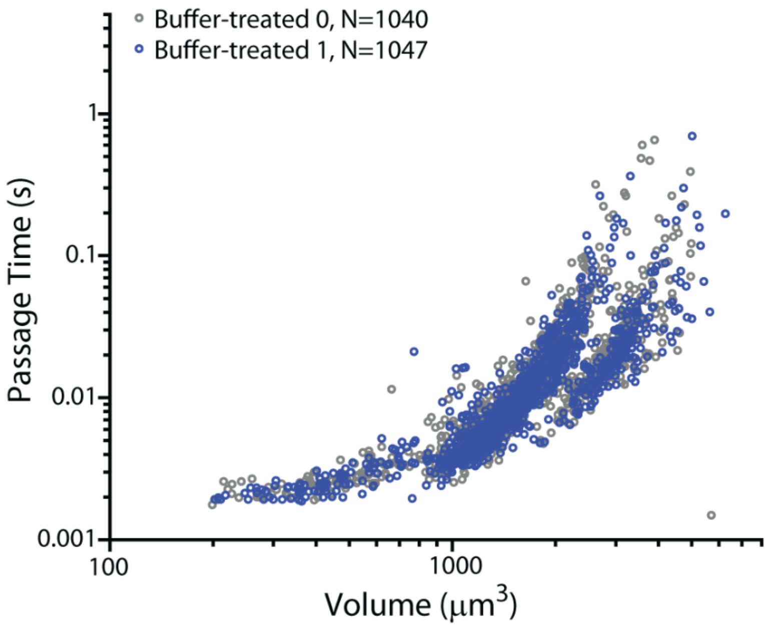

Fig S1. Control versus control
Two control plates of EP5 cells that had been treated with buffer were measured in the SMR. The difference in passage time versus volume between these two buffer-treated samples serves as a control for identifying significant differences in other cell line comparisons.

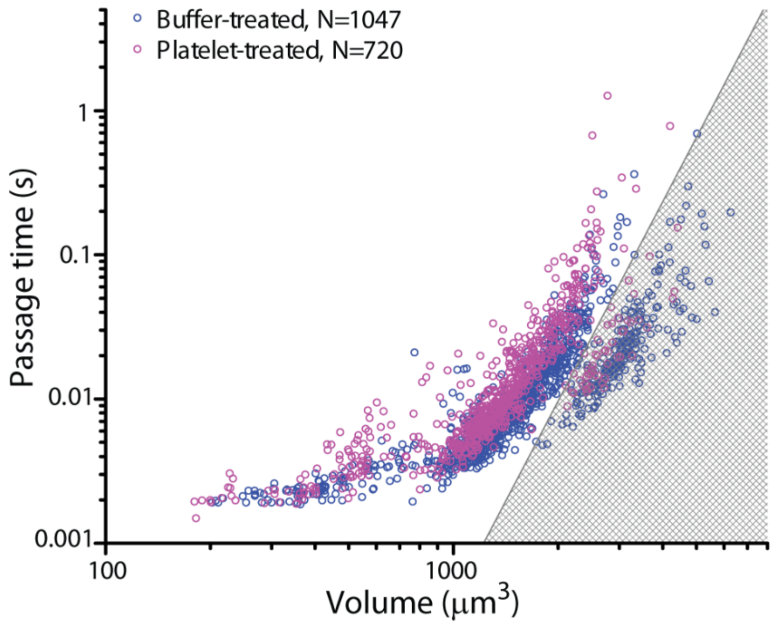

Fig. S2. Removal of putative doublets for passage time ratio analysis
As an example, the same data from Fig. 2E is shown here. To determine the passage time ratio between the two data sets, the shaded region was used to remove the second, larger population of cells, which was presumed to be doublets due to their size.

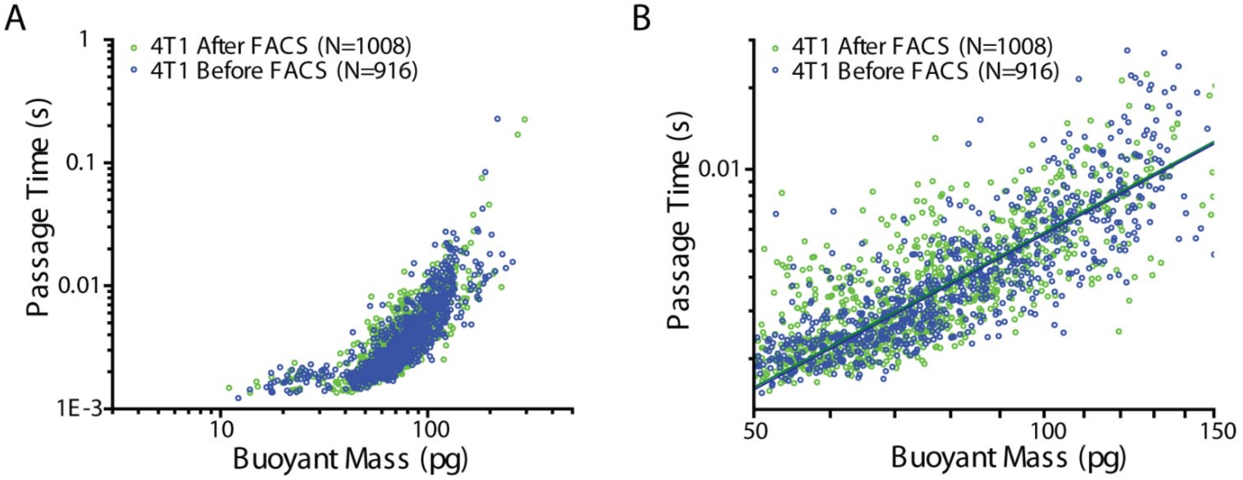

Fig. S3. FACS does not affect passage time characteristic of 4T1-ZSGreen cells
A) The effect of FACS on the passage time versus buoyant mass profile of 4T1-ZSGreen cells was determined by comparing an aliquot of cells that had been run through FACS and one that had not. B) Linear fits were made to the two data sets as shown. The passage time ratio between the cells after FACS and before FACS is 1.02, which is in the range of having no change as measured by the SMR (Fig 2 in main text). The intercept offset between the linear fits to the two data sets is not significantly different ($p = 0.233$).

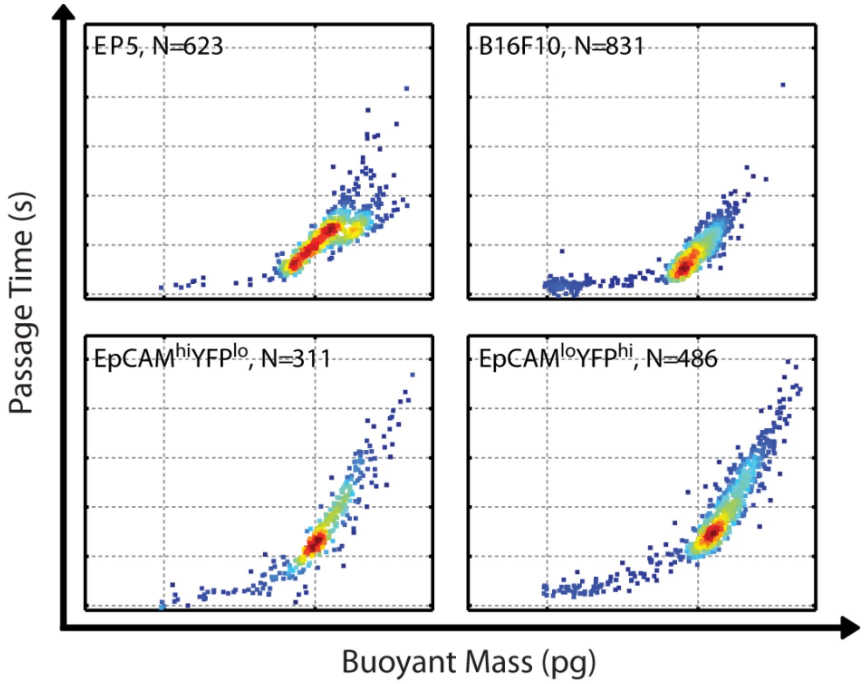

Fig. S4. Passage time versus buoyant mass characteristics of murine tumor cell lines
The mouse tumor cell lines were measured with an applied pressure of 1.5 psi, which is the same condition as the measurements of mouse CTCs in the main text (Fig. 4). Plots are on a log-log scale, with an X-axis ranging from 3 pg to 600 pg, and a Y-axis ranging from 0.008 s to 300 s. Dotted grid lines on the X-axis are at 10 pg and 100pg, while dotted grid lines on the Y-axis are at 0.001 s, 0.01 s, 0.1 s, 1 s, 10 s, and 100 s.

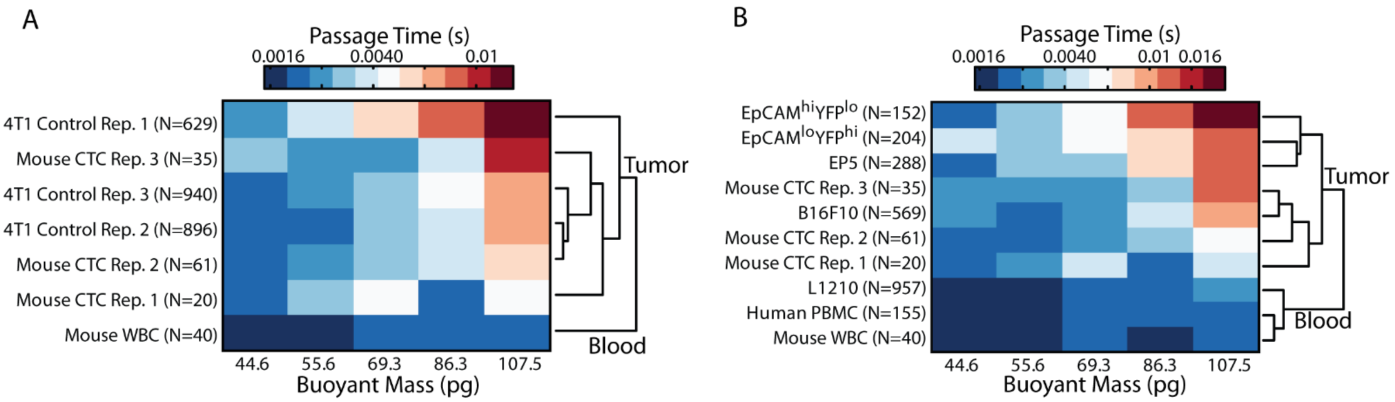

Fig. S5. Each replicate of mouse CTCs clusters with other tumor cell lines.
A) Data from each mouse CTC experiment are used separately in the agglomerative clustering analysis. Each replicate clusters together with control 4T1 cells, and not with BALB/c leukocytes. B) Each replicate of the mouse CTCs clusters together with murine tumor cell lines other than 4T1, rather than blood cells. For both (A) and (B), the buoyant mass range was taken from 40 pg to 120 pg, and the passage time within each bin of buoyant masses is represented by the color of the heat map. The color bar is on a log scale, and the reported buoyant mass bin centers and passage time values are converted from $\log_{10}$ values. The dendrograms depicting the clustering analysis are shown to the right of the heat maps.

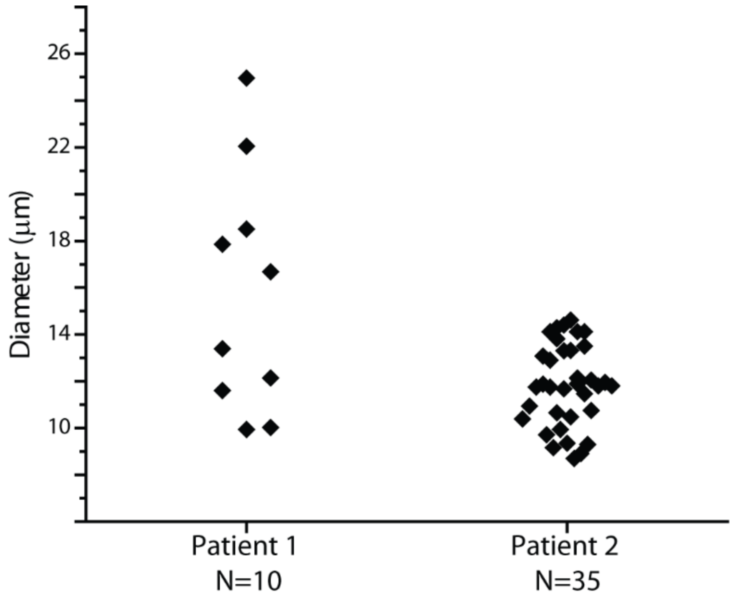

Fig. S6. CTC diameters
CTC diameters were determined as the maximum Feret's diameter of the EpCAM fluorescence signal for each cell.

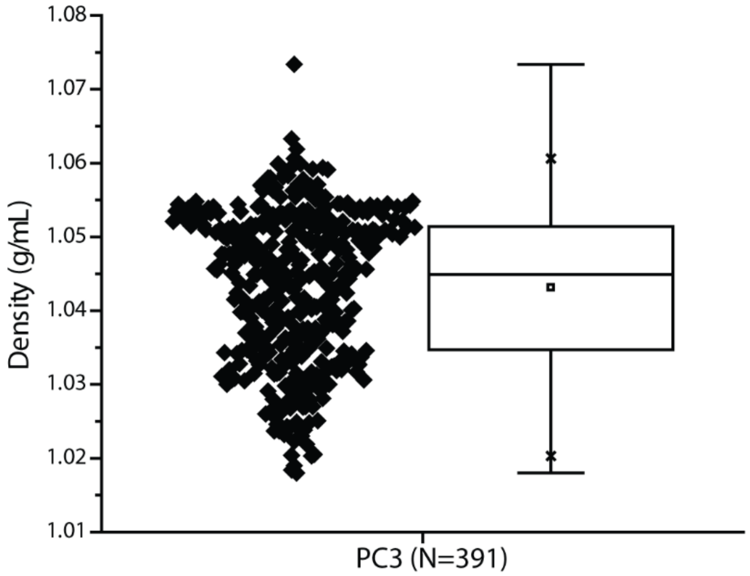

Fig. S7. SMR density measurements of PC3 cells.
The density distribution of PC3 cells measured by the SMR was used to convert CTC diameters to buoyant masses.

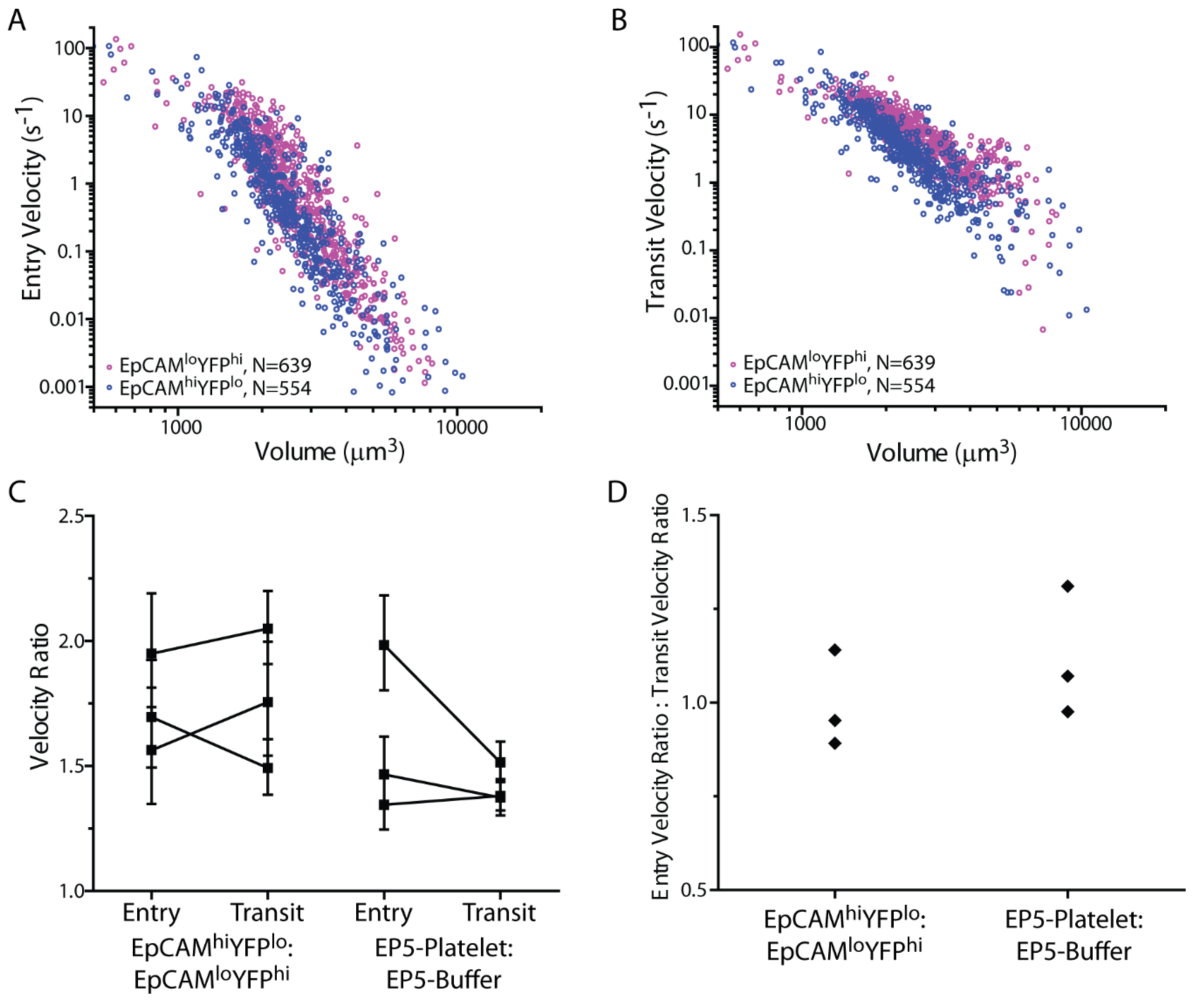

Fig. S8. Entry and transit velocity ratios

A) Entry velocity versus volume for EpCAM^{hi}YFP^{lo} and EpCAM^{lo}YFP^{hi} cells. B) Transit velocity versus volume for EpCAM^{hi}YFP^{lo} and EpCAM^{lo}YFP^{hi} cells. C) Entry and transit velocity ratios for each replicate of experiment are shown, comparing EpCAM^{hi}YFP^{lo} to EpCAM^{lo}YFP^{hi} as well as comparing EP5 cells treated with platelets to those treated with buffer. The velocity ratios are calculated in the same manner as the passage time ratios described in the Supplementary Materials and Methods. The entry and transit velocities change similarly, with neither change dominating the other. D) For each measurement, the entry velocity ratio was divided by the transit velocity ratio to more clearly compare the contribution of each in both cell lines having undergone an EMT or co-incubation with platelets. There is no significant difference between the ratios for the two cases ($p = 0.4$, two-sided Wilcoxon rank-sum test). Also, in both cases the ratios (entry velocity ratio : transit velocity ratio) are not significantly less than 1 ($p = 0.5$ for EpCAM^{hi}YFP^{lo}:EpCAM^{lo}YFP^{hi} and $p = 0.875$ for EP5-Platelet:EP5-Buffer, using a one-sided sign test), indicating that frictional roles do not have a dominant contribution to the changes seen in passage time measurements.

Supplementary Table

Table S1. Methods for measuring single-cell deformability

Technology	Local or Global Measurement	Description	References
Atomic Force Microscopy	Local	• Applies small forces (pN to µN) to locally deform cell's surface<br>• Measures Young's modulus	1–3
Microrheology	Local	• Uses small particles to passively or actively probe viscoelastic properties of cell regions<br>• Magnetic twisting can probe the cell surface	4–8
Micropipette aspiration	Local or Entire cell	• Suction cell partially or fully into micropipette<br>• Cell geometry provides insight into elastic and viscoelastic properties	9,10
Optical Stretcher	Entire cell	• Two laser beams apply stretching force to cell<br>• Cell images provide size and deformation information as measure of compliance	11,12
Hydrodynamic deformation	Entire cell	• Hydrodynamic deformation applied to cells in microfluidic channel<br>• High throughput measurement of aspect ratio or circularity of cell	13–15
Microfluidic constrictions	Entire cell	• Cells are pushed through a constricted channel<br>• Deformability is indicated by amount of time taken to pass through the constriction	16–20

Supplementary Materials and Methods

Cell Culture and Preparation

EP5 and B16F10 cells were cultured in Dulbecco's Modified Eagle's Medium (DMEM), 10% fetal calf serum (FCS), 1% penicillin/streptomycin and 2 mM L-glutamine and obtained as described previously[21]. Cells were detached from the plate using 0.25% Trypsin-EDTA. The MMTV-PyMT tumor cell line was derived from a mammary carcinoma of a *Snail$^{YFP/+}$; MMTV-PyMT* animal. The cells were cultivated in DMEM/F12 (1:1) supplemented with 5% adult bovine serum (Sigma B9433), non-essential amino acids (Invitrogen 11140), and Pen/Strep (Invitrogen 15070). They were then stained with anti-EpCAM antibody directly conjugated to allophycocyanin (APC) fluorophore (Ebioscience 17-5791-82), and sorted by FACS based on EpCAM and Snail^{YFP} expression. The cells were then cultured for 2 or 4 days (passaged once) and then trypsinized (0.08% Trypsin-EDTA), resuspended in culture medium, and measured in the SMR. The 4T1 cells were stably transfected to express ZSGreen as previously described[22], and were cultured in DMEM, 10% fetal bovine serum (FBS), 1% penicillin/streptomycin, 2 mM L-glutamine, 1% non-essential amino acids. MDA-MB231, SKBR3, and PC3-9 cell lines were obtained and cultured as described previously[23]. H1975 cells were obtained previously[24], and cultured in RPMI supplemented with 10% FBS, 1% sodium pyruvate, and 100 IU of penicillin and 100 µg/mL streptomycin. L1210 cells (a gift from Dr. Marc Kirschner) were maintained in suspension in RPMI with 10% fetal bovine serum (FBS), 1% penicillin/streptomysin, 25 mM HEPES. Cells were maintained in incubators at 37°C with 5% CO_2.

Blood Cell Preparation
Whole human blood was purchased from Research Blood Components, LLC (Brighton, MA). Mononuclear cells were separated using Histopaque-1077 (Sigma-Aldrich 10771). Polymorphonuclear lymphocytes were enriched by using Lympholyte-poly (Cedarlane CL5070). Red blood cells were measured by diluting whole blood in PBS with 1% (w/v) Kolliphor P188 (Sigma-Aldrich), since the concentration of red blood cells is orders of magnitude higher than that of other cell types.

Healthy BALB/c mouse blood was obtained via cardiac puncture. Red blood cells were removed by lysis (150 mM NH_4Cl (8.02 g/L), 10 mM $KHCO_3$ (1.00 g/L), 0.1 mM Na_2EDTA, pH 7.23) or Histopaque-1077. To isolate live leukocytes and ensure removal of clots and platelets, the remaining cell solution was stained for CD45 (Biolegend 103121) and CD41 (Biolegend 101319) for 15 min at room temperature after pre-incubation with Fc block (Biolegend 101319). DAPI (Life Technologies D1306) was also used to stain dead cells. After resuspending the cells in PBS with 0.5%FBS and 2mM EDTA, cells were sorted using fluorescence-activated cell sorting (FACS) and the $CD45^{hi}/CD41^{lo}/DAPI^{lo}$ cells were collected for analysis in the SMR.

Buoyant mass to volume conversion
For measurements where the difference between cell types may be subtle, buoyant mass is converted to volume as a metric for size, since the volume of the cell is more consistently related to passage time than is buoyant mass. The single-cell densities of platelet-treated EP5 cells, buffer-treated EP5 cells, $EpCAM^{hi}YFP^{lo}$ MMTV-PyMT cells, and $EpCAM^{lo}YFP^{hi}$ MMTV-PyMT cells were measured on each day of experiment using the SMR as previously described[25]. The density of the culture medium in which the cells were measured was determined by the resonant frequency of the SMR. The volume of the cell was determined by the ratio of the buoyant mass to the difference in the average cell density (typical interquartile range: 0.003-0.007 g/mL) and the fluid density.

Calculating passage time ratio, removing secondary population of cells
The passage time ratios, as shown in Fig. 2C and F, were calculated as previously described[17]. In brief, the passage time versus volume data was plotted on a log-log scale. A line was fit to each data set, having the same slope, but variable intercepts. The difference between the two intercepts corresponds to the $\log_{10}$ of the ratio of the passage times, which is then converted to the actual passage time ratio by exponentiation.

To obtain accurate linear fits, the data was considered within its linear region (on a log-log scale) containing the majority of the cells for analysis, eliminating small particles or debris. Thus, the lines were fit for EP5 cells having volumes between 800 and 3500 μm^3, and for MMTV-PyMT cells greater than 1000 μm^3. As noted in the figure caption (Fig. 2), the EP5 buffer-treated and platelet-treated cells have a second population of cells presumed to be doublets due to their being twice the volume of the majority of the cells in the population. To obtain accurate linear fits, the second population of cells was removed by excluding cells below a given line roughly parallel to the major axis of the second population of cells. The same line was used to remove the second population on both the platelet-treated and buffer-treated cells for all three replicates of the experiment. Fig. S2 shows an example of the removal of the larger population of cells.

Converting imaged cell diameter to buoyant mass
The PC3 prostate cancer cell line was used to estimate the single-cell density of prostate cancer CTCs. PC3 cells were stained for EpCAM and resuspended in PBS with 1% Kolliphor for SMR measurement as would typically be done with patient samples. The single-cell densities of one aliquot of cells were measured in the SMR, while another aliquot was placed in a 24-well plate where they were fixed in 4% paraformaldehyde (PFA) and imaged. From image processing, we found that the maximum Feret's

diameter determined by ImageJ software corresponded well to the volume measured by the SMR. The maximum Feret's diameter found for each CTC is shown in Fig. S6. Since the number of CTCs was low and the density distribution of the PC3 cells in 1% Kolliphor buffer was found to be quite spread (interquartile range: 0.017 g/mL, Fig. S7) compared to typical density measurements, instead of using one set density value to convert all of the CTC diameters to buoyant mass, a simulation was used to randomly assign a density value from the measured distribution to each CTC. Buoyant mass was then calculated by the following equation:

$$Buoyant\ mass = \frac{4}{3}\pi R^3(\rho_{cell} - \rho_{fluid}),$$

where R corresponds to the radius of the imaged cell, ρ_{cell} is the density of the cell, and ρ_{fluid} is the density of the buffer used for measurement. The result is plotted in Fig. 5B and 5E. To determine the number of cells that fell in the buoyant mass range of 50 pg to 100 pg, or greater than 100 pg, the simulation was repeated 1000 times. The average number of cells in each buoyant mass range of interest was calculated and reported in the main text.

References

1. Radmacher, M. Measuring the elastic properties of living cells by the atomic force microscope. *Methods Cell Biol.* **68,** 67–90 (2002).

2. Hansma, H. G. & Hoh, J. H. Biomolecular imaging with the atomic force microscope. *Annu. Rev. Biophys. Biomol. Struct.* **23,** 115–39 (1994).

3. Touhami, A., Nysten, B. & Dufrêne, Y. F. Nanoscale mapping of the elasticity of microbial cells by atomic force microscopy. *Langmuir* **19,** 4539–4543 (2003).

4. Bausch, A. R., Ziemann, F., Boulbitch, A. A., Jacobson, K. & Sackmann, E. Local measurements of viscoelastic parameters of adherent cell surfaces by magnetic bead microrheometry. *Biophys. J.* **75,** 2038–2049 (1998).

5. Tseng, Y., Kole, T. P. & Wirtz, D. Micromechanical mapping of live cells by multiple-particle-tracking microrheology. *Biophys. J.* **83,** 3162–76 (2002).

6. Wang, N., Butler, J. P. & Ingber, D. E. Mechanotransduction across the cell surface and through the cytoskeleton. *Science* **260,** 1124–1127 (1993).

7. Yamada, S., Wirtz, D. & Kuo, S. C. Mechanics of living cells measured by laser tracking microrheology. *Biophys. J.* **78,** 1736–1747 (2000).

8. Valberg, P. A. & Feldman, H. A. Magnetic particle motions within living cells. Measurement of cytoplasmic viscosity and motile activity. *Biophys. J.* **52,** 551–561 (1987).

9. Hochmuth, R. M. Micropipette aspiration of living cells. *J. Biomech.* **33,** 15–22 (2000).

10. Evans, E. & Yeung, A. Apparent viscosity and cortical tension of blood granulocytes determined by micropipet aspiration. *Biophys. J.* **56,** 151–60 (1989).

11. Guck, J., Ananthakrishnan, R., Mahmood, H., Moon, T. J. & Cunningham, C. C. The optical stretcher : a novel laser tool to micromanipulate cells. *Biophys. J.* **81,** 767–784 (2001).

12. Lincoln, B. *et al.* Deformability-based flow cytometry. *Cytometry. A* **59,** 203–9 (2004).

13. Gossett, D. R. *et al.* Hydrodynamic stretching of single cells for large population mechanical phenotyping. *Proc. Natl. Acad. Sci.* **109,** 7630–5 (2012).

14. Tse, H. T. K. *et al.* Quantitative diagnosis of malignant pleural effusions by single-cell mechanophenotyping. *Sci. Transl. Med.* **5,** 212ra163 (2013).

15. Otto, O. *et al.* Real-time deformability cytometry: on-the-fly cell mechanical phenotyping. *Nat. Methods* **12,** (2015).

16. Hou, H. W. *et al.* Deformability study of breast cancer cells using microfluidics. *Biomed. Microdevices* **11,** 557–64 (2009).

17. Byun, S. *et al.* Characterizing deformability and surface friction of cancer cells. *Proc. Natl. Acad. Sci.* **110,** 7580–5 (2013).

18. Rosenbluth, M. J., Lam, W. A. & Fletcher, D. A. Analyzing cell mechanics in hematologic diseases with microfluidic biophysical flow cytometry. *Lab Chip* **8,** 1062–70 (2008).

19. Zheng, Y., Shojaei-Baghini, E., Azad, A., Wang, C. & Sun, Y. High-throughput biophysical measurement of human red blood cells. *Lab Chip* **12,** 2560 (2012).

20. Zhang, W. *et al.* Microfluidics separation reveals the stem-cell-like deformability of tumor-initiating cells. *Proc. Natl. Acad. Sci. U. S. A.* **109,** 18707–12 (2012).

21. Labelle, M., Begum, S. & Hynes, R. O. Direct signaling between platelets and cancer cells induces an epithelial-mesenchymal-like transition and promotes metastasis. *Cancer Cell* **20,** 576–90 (2011).

22. Lamar, J. M. *et al.* The Hippo pathway target, YAP, promotes metastasis through its TEAD-interaction domain. *Proc. Natl. Acad. Sci.* **109,** E2441–50 (2012).

23. Ozkumur, E. *et al.* Inertial focusing for tumor antigen-dependent and -independent sorting of rare circulating tumor cells. *Sci. Transl. Med.* **5,** 179ra47 (2013).

24. Yauch, R. L. *et al.* Epithelial versus mesenchymal phenotype determines in vitro sensitivity and predicts clinical activity of erlotinib in lung cancer patients. *Clin. Cancer Res.* **11,** 8686–8698 (2005).

25. Grover, W. H. *et al.* Measuring single-cell density. *Proc. Natl. Acad. Sci.* **108,** 10992–6 (2011).
